# Injectable Poloxamer and Hyaluronic Acid Hydrogel for Sustained Co-Delivery of Dexamethasone and Lidocaine Ameliorates Neuropathic Pain

**DOI:** 10.34133/bmr.0373

**Published:** 2026-05-28

**Authors:** Yanting Liu, Seungwoon Baik, Trung Nhan Vo, Songzi Zhang, Boram Kim, Tae-Keun Ahn, Inbo Han, Dong Keun Han

**Affiliations:** ^1^ Department of Neurosurgery, CHA Bundang Medical Center, CHA University, Seongnam-si 13496, Republic of Korea.; ^2^ ORANDBIO Co. Ltd., Uiwang-si, Gyeonggi-do16108, Republic of Korea.; ^3^ Department of Biomedical Science, CHA University, Seongnam-si 13488, Republic of Korea.; ^4^ College of Basic Medical Sciences, Jilin University, Changchun 130021, China.; ^5^ Department of Orthopedic Surgery, CHA Bundang Medical Center, CHA University, Seongnam-si 13496, Republic of Korea.

## Abstract

Neuropathic pain is a debilitating condition driven by complex neuro–immune–glial interactions and central sensitization. Current pharmacological interventions, such as opioids and ion channel blockers, are often limited by short half-lives, systemic toxicity, and tolerance. To overcome these barriers, we developed an injectable, thermosensitive hydrogel system based on poloxamer and hyaluronic acid (PLX/HA) for the sustained, localized co-delivery of dexamethasone (Dex) and lidocaine (Lid). In vitro characterization demonstrated that HA incorporation optimized the hydrogel’s microstructural porosity, enabling the sustained release of bioactive agents for up to 28 d. At the cellular level, the system marked enhanced neuronal survival under inflammatory stress by modulating the Bax/Bcl-2 apoptotic pathway and suppressing nuclear factor κB (NF-κB) nuclear translocation and cyclooxygenase-2 (COX-2) expression. In a rat model of chronic constriction injury (CCI), the Dex/Lid@PLX/HA formulation provided sustained improvement in mechanical allodynia and thermal hyperalgesia, with greater efficacy than the solution and standard gel control groups at multiple time points. Mechanistically, the therapeutic effects were accompanied by attenuation of TRPV1, CGRP, and SGC activation-related signals, suggesting suppression of peripheral nociceptive sensitization and attenuation of DRG neurodegenerative pathology, including reduced Nageotte nodule-like changes. Furthermore, the PLX/HA system was associated with reduced proinflammatory macrophage infiltration and an increased CD163-positive macrophage signal, suggesting a more reparative local immune microenvironment. This study presents a localized co-delivery strategy with analgesic and anti-inflammatory effects for neuropathic pain management and peripheral nerve protection.

## Introduction

Neuropathic pain, a debilitating chronic pain condition resulting from direct injury or pathological dysfunction of the somatosensory nervous system, remains one of the most intractable forms of pain. It is clinically characterized by spontaneous pain, hyperalgesia, and allodynia. This condition is frequently observed in diabetic peripheral neuropathy, postherpetic neuralgia, and traumatic lesions of the peripheral or central nervous system, such as brachial plexus or spinal cord injury [1,2]. While current management strategies rely heavily on the systemic administration of tricyclic antidepressants, gabapentinoids, opioids, and sodium channel blockers, these therapies face formidable challenges [3–7]. Pharmacokinetically, agents like lidocaine (Lid) and dexamethasone (Dex) have extremely short half-lives and undergo rapid metabolism, making it difficult to maintain effective therapeutic concentrations at the nerve lesion without frequent or high-dose administration. Systemically, such regimens pose severe toxicity risks; for instance, Lid overdose can precipitate cardiac arrhythmias and central nervous system seizures, while long-term glucocorticoid use is associated with hyperglycemia, immunosuppression, osteoporosis, and suppression of the hypothalamic–pituitary–adrenal axis [8,9]. Furthermore, the chronic use of opioids is severely limited by tolerance, dependence, and the global addiction crisis. Consequently, the development of a localized delivery system capable of targeted, sustained drug release with good biocompatibility is critical to overcoming these therapeutic bottlenecks [10–12].

The pathogenesis of neuropathic pain extends beyond simple neuronal electrophysiological abnormalities; it is an intricate cascade involving complex interactions among neurons, immune cells, and glial cells [13]. Peripheral nerve injury triggers Wallerian degeneration and the release of damage-associated molecular patterns, which stimulate peripheral nociceptors. The transient receptor potential vanilloid 1 (TRPV1) channel plays a pivotal role in this process [14]. Injury leads to an up-regulation of TRPV1 expression in dorsal root ganglion (DRG) neurons, effectively lowering the activation threshold of nociceptors. The activation of TRPV1 not only induces calcium influx, leading to neuronal depolarization, but also triggers the release of neuropeptides such as calcitonin gene-related peptide (CGRP) from nerve terminals [15,16]. CGRP, acting as a potent vasodilator and neurotransmitter, further promotes neurogenic inflammation, establishing a positive feedback loop that maintains sustained nociceptive input. Following injury, nerve tissue recruits circulating monocytes that differentiate into macrophages and accumulate at the lesion site. In the early phase, these macrophages predominantly polarize into the proinflammatory M1 phenotype, secreting cytokines such as tumor necrosis factor-α (TNF-α), interleukin-1β (IL-1β), and IL-6 [17,18]. These factors directly sensitize neuronal receptors and enhance sodium channel activity, thereby exacerbating pain. However, pain resolution and nerve repair depend on the transition of macrophages toward the anti-inflammatory, pro-reparative M2 phenotype. Chronic neuropathic pain is often characterized by a persistent M1/M2 imbalance, where sustained M1-driven inflammation impedes tissue repair. In the DRG, the somata of sensory neurons are tightly enveloped by SGCs. Upon nerve injury, SGCs become activated, a state manifested by the up-regulation of glial fibrillary acidic protein (GFAP) and cell proliferation [19]. This reactive gliosis leads to pathological neuron–glia coupling, facilitating the abnormal spread of excitatory signals between neurons. Furthermore, the interaction between SGCs and phagocytes may lead to the formation of Nageotte nodules, and residual cellular clusters formed after neuronal death, which are recognized as a critical pathological hallmark of neurodegeneration and the persistence of chronic pain.

Given the complex pathology of neuropathic pain, monotherapy often fails to achieve adequate relief. The co-delivery of Dex and Lid proposed herein is based on a clear logic of synergistic potentiation established in pharmacology [20–22]. Dex is selected for its potent ability to suppress the synthesis of proinflammatory cytokines, a mechanism critical for dampening the neuroinflammatory storm. Furthermore, Dex is known to down-regulate the expression of cationic channels in injured neurons, consistent with our observation of reduced TRPV1 expression. Complementing this, Lid is utilized to block voltage-gated sodium channels, thereby suppressing ectopic discharges [23,24]. Beyond anesthesia, recent evidence suggests that Lid can modulate the reactivity of SGCs, aligning with our goal to interrupt peripheral-to-central sensitization. However, the short half-lives of these agents necessitate a sustained-release delivery strategy to maintain effective local concentrations.

To address these pharmacokinetic limitations and maximize therapeutic outcomes, we engineered a thermosensitive composite hydrogel system integrating poloxamer (PLX) and hyaluronic acid (HA) [25,26]. This design leverages the unique synergy of its constituents: The thermosensitive PLX base allows for injectable in situ gelation to ensure precise perineural retention, while the hydrogel network better mimics the extracellular matrix (ECM) to enhance mechanical stability and support tissue structural integrity. Crucially, HA serves a dual purpose by optimizing the hydrogel’s microarchitecture to facilitate sustained drug release and actively remodeling the immune microenvironment; specifically, our data demonstrate that HA drives a phenotypic shift in macrophages toward the reparative M2 state, counteracting proinflammatory signaling. In this study, using a rat CCI model, we systematically evaluated the efficacy of this Dex/Lid-loaded PLX@HA platform. By characterizing its sustained analgesic effects and dissecting its impact on the TRPV1–CGRP signaling axis and pathological satellite glial cell activation, we explored the possible basis for its therapeutic effects in neuropathic pain management.

## Materials and Methods

### Preparation and characterization of the functional hydrogel

Three formulations were prepared and designated as Dex/Lid@PBS, Dex/Lid@PLX, and Dex/Lid@PLX/HA. Lid hydrochloride and Dex were dissolved in phosphate-buffered saline (PBS) solution to obtain final concentrations of 0.3% (w/v) Lid and 5 mg/ml Dex, and this solution was used as the Dex/Lid@PBS group. For the Dex/Lid@PLX group, Pluronic F127 and F68 were mixed in PBS at a weight ratio of 8:2 to form a PLX-based hydrogel with a final polymer concentration of 30% (w/v) containing 0.3% Lid and 5 mg/ml Dex. Dex/Lid@PLX/HA was prepared by mixing the PLX hydrogel (F127:F68 = 8:2, 30% w/v) with HA gel at a volume ratio of 3:1 (Pluronic:HA), resulting in the same final drug concentrations (0.3% Lid and 5 mg/ml Dex). Dex/Lid@PBS, Dex/Lid@PLX, and Dex/Lid@PLX/HA formulations were visually examined at 25 and 37 °C to evaluate their temperature-dependent sol–gel transition behavior. For morphological observation, the samples were freeze-dried and directly imaged using a scanning electron microscope (SEM; S-4800, HITACHI. Japan). Rheological properties, including storage modulus (*G*′), loss modulus (*G*″), and tan δ, were analyzed at 37 °C using a strain-controlled rheometer (Anton Paar, Graz, Austria). Drug release profiles were evaluated at 37 °C using a transwell-type setup, where each formulation was placed on an insert and the released drug diffused into PBS in the lower chamber. At predetermined time points, aliquots of the PBS were collected and analyzed by high-performance liquid chromatography (HPLC) (Thermo Scientific, MA, USA). The chromatographic conditions were as follows: mobile phase consisting of acetonitrile and 0.1% formic acid in water at a ratio of 70:30 (v/v), pH 2.8, detection wavelength 350 nm, column temperature 35 °C, flow rate 0.8 ml/min, and a total running time of 15 min. To evaluate the antioxidant activity, a DPPH free radical scavenging assay was performed. Each sample was incubated with 1 ml of 2,2-diphenyl-1-picrylhydrazyl (DPPH) solution (25 mM in ethanol) at 37 °C for 24 h in the dark under gentle shaking. After incubation, the absorbance of the supernatant was measured at 516 nm using a microplate reader (SpectraMax M2, Molecular Devices, USA).

### Animals and housing

Ten-week-old male Sprague–Dawley rats, weighing between 200 and 220 g, were obtained from Koatech (Korea) and maintained under standardized conditions. The animals were housed in an environmentally controlled facility at 24 ± 3 °C with a relative humidity of 55% to 65% and a 12-h light/dark cycle, with food and water provided ad libitum. Following arrival, the rats were acclimatized for 1 week prior to any experimental procedures.

### Ethical considerations

All animal procedures were performed in strict accordance with the guidelines set forth by the Institutional Animal Care and Use Committee (IACUC) of CHA University (IACUC230018). Every effort was made to minimize animal suffering and to use the fewest number of animals necessary to achieve statistically meaningful results.

### Experimental groups and study design

Rats were randomly divided into 5 groups (*n* = 6 per group): (a) an uninjured Naive group, (b) a CCI group without treatment, (c) a treatment group receiving Dex and Lid in PBS, (d) a treatment group receiving Dex/Lid in a gel formulation, and (e) a treatment group receiving Dex/Lid in a HA-enhanced PLX hydrogel formulation (Dex/Lid@PLX/HA). This design enabled a comparative evaluation of the analgesic and neuroprotective efficacy of the different drug formulations.

### Surgical procedures and neuropathic pain induction

To elucidate the pathophysiological mechanisms and evaluate such novel interventions, robust animal models are indispensable; among them, the chronic constriction injury (CCI) model in rats is widely employed as it faithfully replicates key features of peripheral neuropathy, including persistent thermal hyperalgesia, mechanical allodynia, and associated neuroinflammatory changes [27,28]. To induce neuropathic pain, rats were anesthetized by intraperitoneal injection of tiletamine and zolazepam (Zoletil, 50 mg/kg; Virbac Laboratories) combined with xylazine (Rompun, 10 mg/kg; Bayer). Under aseptic conditions, a small incision was made along the lateral aspect of the right thigh to expose the sciatic nerve. The nerve was then loosely ligated at 4 sites using 4-0 silk sutures at 1-mm intervals, until a slight twitching of the hindlimb muscles was observed, thereby confirming the successful induction of the CCI model. Immediately after ligation, a 100-μl injection of the assigned formulation was administered locally at the lesion site. Specifically, the PBS group received Dex/Lid in solutions, while the Gel and PLX/HA groups were treated with their respective formulations. The surgical wound was then closed with 4-0 silk sutures, and the animals were monitored daily until euthanasia 28 d post-surgery.

### Behavioral assessments

Two complementary behavioral tests were conducted to evaluate pain sensitivity following injury. Mechanical sensitivity was quantified using an electro-von Frey device (Ugo Basile, model 37000-007). Rats were placed in a plastic observation chamber with a mesh floor and allowed a 10-min acclimatization period. The paw withdrawal threshold (PWT), measured in grams and averaged over 3 consecutive trials, was recorded 1 d prior to injury and on post-injury days 1, 3, 7, 14, 21, and 28. Thermal sensitivity was assessed using a Hot Plate apparatus (Ugo Basile, model 35150-001) set at 52 °C. After a 10-min acclimatization period in a quiet chamber, each rat was placed on the heated surface, and the latency to display a nociceptive response, such as paw lifting, licking, jumping, or 2 consecutive steps, was recorded. To prevent tissue damage, a cutoff time of 20 s was strictly enforced. Testing was conducted at baseline and at the same post-injury time points as the mechanical assessments.

### Tissue collection and histological processing

At 28 d post-injury, the rats were deeply anesthetized and transcardially perfused with 0.9% saline followed by 4% paraformaldehyde (PFA) in PBS. The spinal cord (L4–L5 region), DRG, and the right sciatic nerve were carefully harvested. Tissues were postfixed overnight in 4% PFA and then processed for paraffin embedding. Sciatic nerve samples were fixed in 10% formalin for 72 h, dehydrated through graded ethanol series, cleared in xylene, and sectioned into 3-μm-thick slices. These sections were subsequently stained with hematoxylin and eosin (H&E) according to standard protocols, facilitating the evaluation of tissue morphology and the extent of inflammatory infiltration.

### Immunofluorescence analysis

For immunofluorescence studies, paraffin-embedded tissue sections were first deparaffinized in xylene and rehydrated through graded ethanol washes. Antigen retrieval was performed by incubating the sections in tris-EDTA buffer in a water bath. Nonspecific binding was minimized by incubating the sections with 1% bovine serum albumin (BSA) in PBS for 30 min at room temperature. The sections were then incubated overnight at 4 °C with the following primary antibodies: TRPV1 (Alomone Labs, catalog number ACC-030-GP), Iba-1 (Abcam, catalog number ab5076), NeuN (Abcam, catalog number ab104224), CD68 (Abcam, catalog number ab31630), CD163 (Abcam, catalog number ab182422), CGRP (Abcam, catalog number ab47027), GFAP (Millipore, catalog number MAB360), and NF200 (Abcam, catalog number ab8135). After thorough washing with PBS, sections were incubated for 1 h at room temperature with species-specific secondary antibodies conjugated to fluorophores, including goat anti-mouse Alexa Fluor 488, goat anti-mouse Alexa Fluor 568, donkey anti-goat Alexa Fluor 568, donkey anti-rabbit Alexa Fluor 568, and donkey anti-rabbit Alexa Fluor 488. Nuclei were counterstained with 4′,6-diamidino-2-phenylindole (DAPI) (Vector Laboratories, USA). Fluorescence images were captured using a Zeiss Axio Scan Z1 digital slide scanner.

Fluorescence and histological images were acquired using a Zeiss Axio Scan Z1 digital slide scanner (Carl Zeiss, Germany) and analyzed using Zen software (Zen 3.1 Blue edition). Quantification was performed under identical acquisition settings and using comparable regions of interest across groups. Positive signal areas or fluorescence intensity was measured from representative, nonoverlapping fields and normalized as indicated in the corresponding figure legends. For DAPI-normalized analyses, fluorescence signals were normalized to the DAPI-positive area. For ratio-based analyses, values were calculated relative to the corresponding reference marker within the same region. Data from histological and immunofluorescence staining were processed using the same analysis workflow across all groups.

### Statistical analysis

Statistical analyses were performed using GraphPad Prism 9.0 (GraphPad Software, San Diego, CA, USA). Behavioral data, including von Frey test (VFT) and hot plate test (HPT), were analyzed using 2-way repeated-measures analysis of variance (ANOVA) followed by Tukey’s multiple-comparisons test. Histological, immunofluorescence, and other endpoint data were analyzed using one-way ANOVA followed by Bonferroni’s or Tukey’s post hoc test, as appropriate. A *P* value of <0.05 was considered statistically significant. Statistical significance was indicated as follows: ns, not significant; *P < 0.05; **P < 0.01; ***P < 0.001; and ****P < 0.0001.

## Results and Discussion

### Characterization of the functional hydrogel

**Fig. 1.**
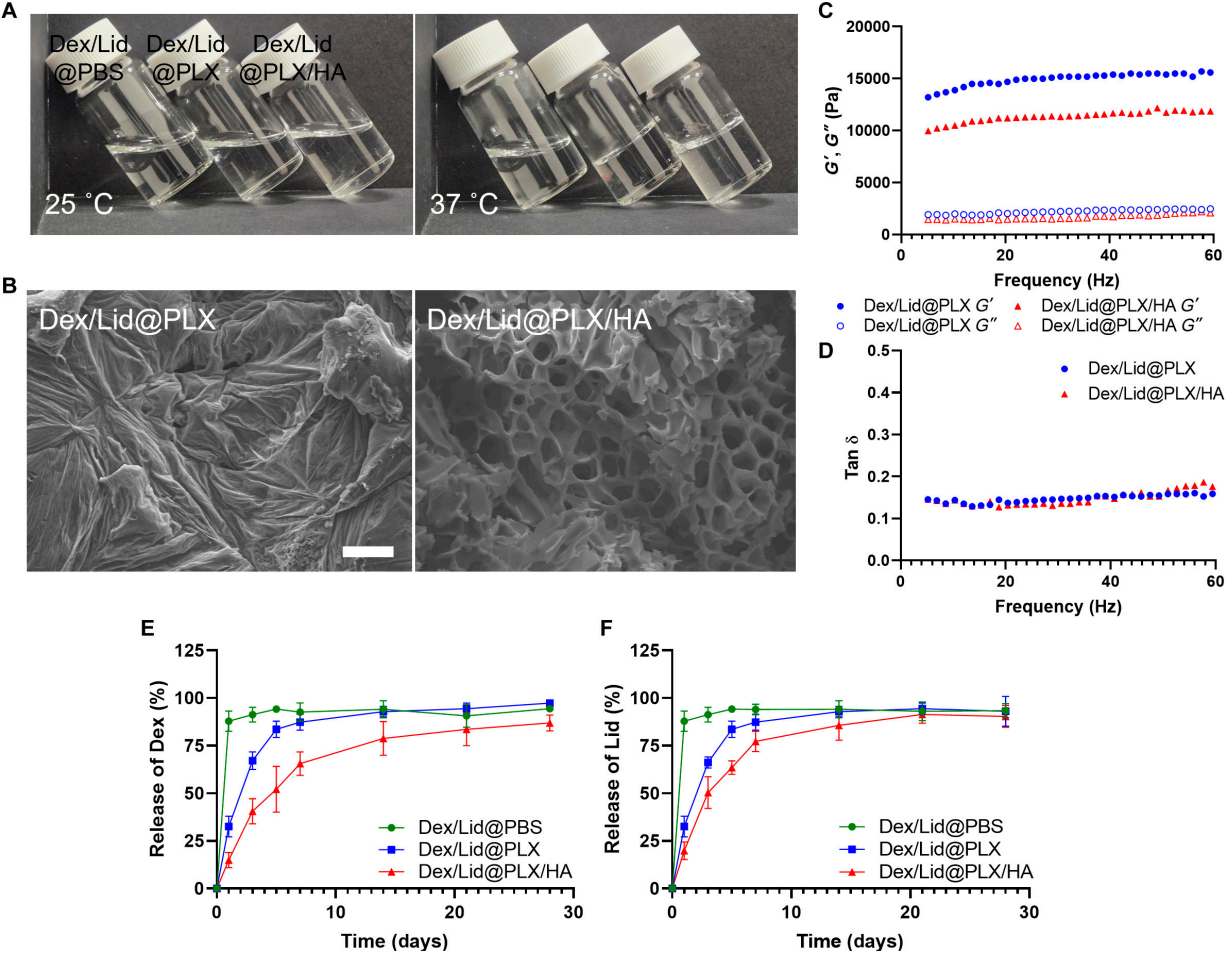
Characterization of the thermosensitive hydrogel system. (A) Representative photographs of Dex/Lid@PBS, Dex/Lid@PLX, and Dex/Lid@PLX/HA at 25 °C (left) and 37 °C (right), demonstrating the temperature-dependent sol–gel transition. (B) SEM micrographs showing the microstructure of Dex/Lid@PLX and Dex/Lid@PLX/HA hydrogels (scale bar, 50 μm). (C and D) Rheological properties of the hydrogels at 37 °C. (C) Storage modulus (*G*′) and loss modulus (*G*″) and (D) damping factor (tan δ) as a function of frequency, obtained from frequency sweep measurements. (E and F) In vitro cumulative release profiles of (E) dexamethasone (Dex) and (F) lidocaine (Lid) in PBS at 37 °C over 28 d. Data are presented as mean ± SD (*n* = 3).

To satisfy the clinical requirements for minimally invasive injection and precise in situ retention, the rheological behavior of the formulations was evaluated. Macroscopically, while Dex/Lid@PBS remained a liquid sol at all temperatures, both Dex/Lid@PLX and Dex/Lid@PLX/HA exhibited a sharp thermosensitive sol–gel transition (Fig. 1A). They existed as low-viscosity sols at room temperature (25 °C), facilitating injection, and rapidly converted into stable semi-solid gels at body temperature (37 °C). This transition is driven by the dense packing of PLX micelles and the hydrophobic dehydration of poly(propylene oxide) (PPO) blocks, ensuring the formation of a cohesive drug depot around the nerve [25].

SEM revealed that HA incorporation fundamentally altered the gel microstructure (Fig. 1B). Unlike the dense, sheet-like morphology of Dex/Lid@PLX—which may hinder solute transport—Dex/Lid@PLX/HA displayed a highly porous, interconnected network. This increased porosity results from the steric hindrance of long-chain HA molecules intercalating between PLX micelles, creating hydrophilic channels. Crucially, this architecture supports the permeation of oxygen and glucose essential for nerve metabolism while simultaneously providing a physical tortuosity barrier for sustained drug diffusion [29]. Rheological quantification at 37 °C confirmed a stable elastic gel state for both formulations, characterized by storage moduli (*G*′) consistently exceeding loss moduli (*G*″) and tan δ values well below 1 (Fig. 1C and D). Notably, the addition of HA slightly reduced the gel stiffness. Biomechanically, this improved compliance is advantageous; the softer matrix better mimics the low elastic modulus of Naive peripheral nerve tissue (approximately 0.5 to 50 kPa), thereby minimizing the risk of mechanical compression or irritation at the bio-interface [30,31].

Consistent with these structural findings, in vitro release profiles (Fig. 1E and F) showed that while Dex/Lid@PBS exhibited a rapid “burst release” (>85% within 24 h), Dex/Lid@PLX/HA achieved the most sustained kinetics. Only ~60% of Dex and ~75% of Lid were released by day 7, confirming that the HA-enhanced network effectively retards drug diffusion to extend the therapeutic window.

### In vitro biocompatibility, anti-apoptotic, and anti-inflammatory evaluation

**Fig. 2.**
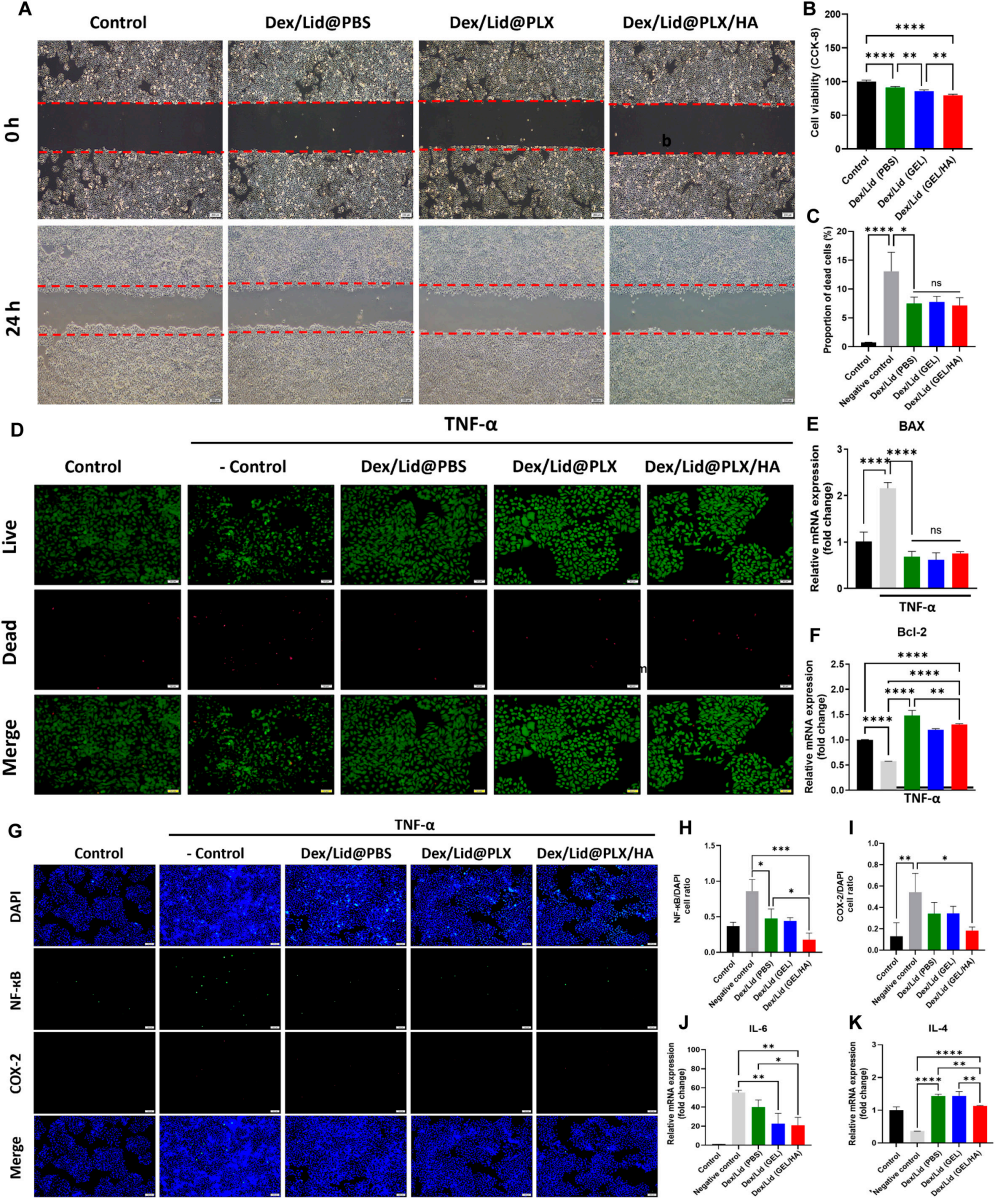
In vitro evaluation of biocompatibility, anti-apoptosis, and anti-inflammatory effects of functional hydrogels on SH-SY5Y cells. (A) Representative optical microscopy images of the wound healing assay at 0 and 24 h (scale bar, 200 μm). (B) Quantitative analysis of cell viability via Cell Counting Kit-8 (CCK-8) assay. (C) Quantitative analysis of the proportion of dead cells. (D) Representative fluorescence images of Live (green)/Dead (red) staining after TNF-α (50 ng/ml) stimulation for 24 h (scale bar, 50 μm). (E and F) Relative mRNA expression levels of apoptosis-related genes (E) BAX and (F) Bcl-2 determined by qRT-PCR. (G) Representative immunofluorescence images showing the expression of NF-κB (green) and COX-2 (red). Nuclei were counterstained with DAPI (blue) (scale bar, 100 μm). (H and I) Quantitative analysis of NF-κB and COX-2 fluorescence signals normalized to the DAPI-positive area. (J and K) Relative mRNA expression levels of (J) IL-6 and (K) IL-4 determined by qRT-PCR. Data are presented as mean ± SD. *P < 0.05, **P < 0.01, ***P < 0.001, and ****P < 0.0001.

The biocompatibility of the hydrogel system was first evaluated using wound healing and cell viability assays. As shown in Fig. 2A, SH-SY5Y cells in all drug-treated groups exhibited migration patterns comparable to the untreated control over 24 h, indicating that the formulations do not impair cellular motility. Although quantitative analysis (Fig. 2B) revealed a statistically significant, albeit minor, reduction in metabolic activity in the drug-loaded groups compared to the control, cell viability remained consistently above 80% across all formulations, confirming an acceptable safety profile for neural application.

We next assessed the cytoprotective capacity of the system under inflammatory stress. Live/Dead staining (Fig. 2D) demonstrated that TNF-α stimulation (negative control) induced substantial cytotoxicity, resulting in a dead cell proportion of approximately 13% (Fig. 2C). In contrast, treatment with Dex/Lid formulations, regardless of the vehicle (PBS, PLX, or PLX/HA), significantly attenuated cytotoxicity, reducing the proportion of dead cells to approximately 7.5%. To elucidate the underlying molecular mechanism, we quantified apoptosis-related gene expression via quantitative reverse transcription polymerase chain reaction (qRT-PCR) (Fig. 2E and F). While TNF-α challenge dramatically up-regulated the proapoptotic marker BAX (Fig. 2E) and down-regulated the anti-apoptotic marker Bcl-2 (Fig. 2F), all Dex/Lid-containing groups effectively reversed this transcriptional shift. Notably, no significant differences in gene expression were observed among the drug-treated groups, suggesting that the hydrogel matrix successfully releases bioactive drugs capable of counteracting cytokine-induced apoptosis within this short-term observation window.

To further elucidate the anti-inflammatory mechanism, we investigated the nuclear factor κB (NF-κB) signaling pathway. Immunofluorescence analysis (Fig. 2G) confirmed that TNF-α stimulation triggered robust NF-κB nuclear translocation and the downstream up-regulation of cyclooxygenase-2 (COX-2). Quantitative analysis (Fig. 2H and I) revealed that while all drug-treated groups attenuated NF-κB fluorescence (Fig. 2H), Dex/Lid@PBS and Dex/Lid@PLX failed to significantly suppress COX-2 expression compared to the negative control (*P >* 0.05; Fig. 2I). This suggests that transient drug exposure is insufficient to counteract persistent inflammatory signaling. In contrast, Dex/Lid@PLX/HA significantly inhibited both NF-κB translocation and COX-2 expression (*P* < 0.05), indicating a stronger anti-inflammatory effect than the PBS and PLX groups under the present experimental conditions. Given that our in vitro assay is specifically designed to evaluate neuroinflammation and cytotoxicity rather than action potential-mediated pain transmission, these profound anti-inflammatory outcomes can be primarily attributed to the potent glucocorticoid action of the released Dex. The specific contribution of Lid, which primarily targets voltage-gated sodium channels to inhibit nociceptive signaling, is more functionally captured in the subsequent in vivo behavioral assessments.

To provide additional evidence of the anti-inflammatory response, we analyzed the gene expression of key cytokines (Fig. 2J and K). While Dex/Lid@PBS failed to reverse the TNF-α-induced up-regulation of the proinflammatory cytokine IL-6 (*P >* 0.05), both hydrogel formulations significantly down-regulated IL-6, with Dex/Lid@PLX/HA achieving the most pronounced reduction (*P* < 0.01; Fig. 2J). Regarding IL-4 expression (Fig. 2K), the PBS and PLX groups showed higher transcript levels under TNF-α stimulation, whereas the Dex/Lid@PLX/HA group maintained IL-4 expression closer to the untreated control level. Because this assay was performed in SH-SY5Y cells rather than immune cells, the biological significance of the IL-4 change should be interpreted cautiously. In the present study, IL-4 was therefore considered an exploratory marker of the overall transcriptional response to inflammatory stress rather than direct evidence of macrophage-related immune modulation.

### PLX/HA formulation alleviates thermal hyperalgesia and mechanical allodynia

**Fig. 3.**
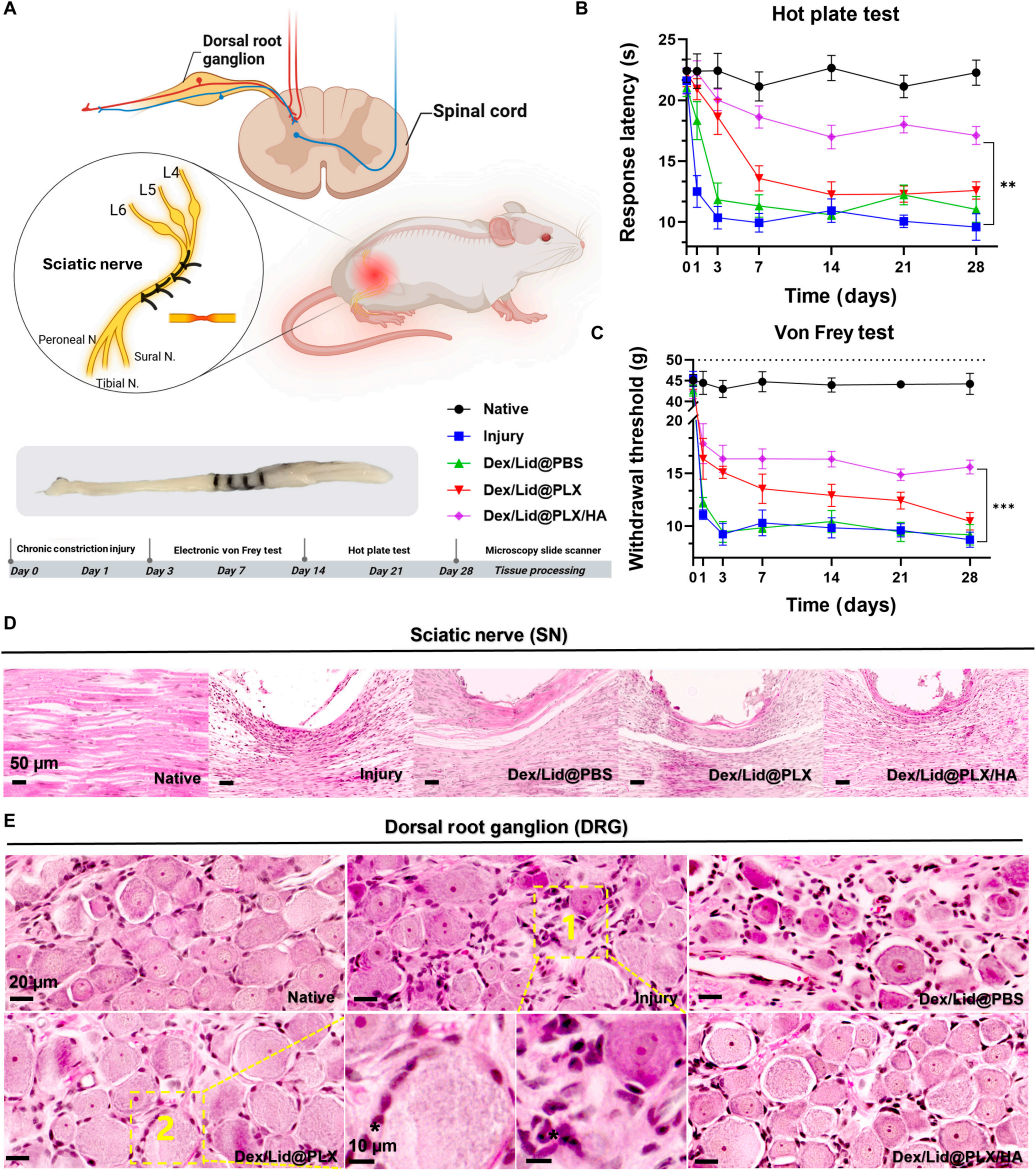
Analgesic efficacy and neuroprotective evaluation of Dex/Lid-loaded hydrogels in a rat CCI model. (A) Schematic of the experimental design and timeline. The CCI model was established via sciatic nerve ligation, followed by treatment with Dex/Lid@PBS, Dex/Lid@PLX, or Dex/Lid@PLX/HA. (B and C) Time-course assessment of (B) thermal hyperalgesia (hot plate test) and (C) mechanical allodynia (von Frey test). Dex/Lid@PLX/HA treatment showed sustained attenuation of nociceptive sensitization (**P < 0.01, ***P < 0.001). (D) H&E staining of the sciatic nerve at day 28. Dex/Lid@PLX/HA preserved axonal architecture and minimized inflammatory infiltration compared to Injury and PBS groups. Scale bar, 50 μm. (E) Histopathology of the dorsal root ganglion (DRG). Scale bars, 20 μm (overview) and 10 μm (insets).

The experimental workflow and CCI model establishment are schematically illustrated in Fig. 3A. To replicate the pathological features of peripheral neuropathy, the CCI model was induced by placing 4 loose ligatures around the common sciatic nerve (originating from the L4–L6 spinal segments) to cause partial nerve compression. Following surgery (day 0), rats were randomized into 5 cohorts: Naive (healthy control), Injury (untreated), Dex/Lid@PBS, Dex/Lid@PLX, and Dex/Lid@PLX/HA. Therapeutic formulations were administered via a single local injection at the ligation site immediately post-surgery. To evaluate longitudinal therapeutic efficacy, behavioral sensitivities were monitored via electronic VFT and HPT at predetermined intervals (days 1, 3, 7, 14, 21, and 28), culminating in tissue harvest for histological and immunofluorescence analysis on day 28.

Parallel assessment of thermal nociception using the HPT (Fig. 3B) corroborated these findings. While baseline latencies were comparable across groups (*P >* 0.9), CCI rats exhibited rapid thermal hyperalgesia starting day 1 (Naive versus Injury: mean diff. = 9.89 s, *P* = 0.0032). Treatment with Dex/Lid@PLX/HA significantly mitigated this hypersensitivity, increasing response latency by 9.68 s compared to the injury group at day 3 (*P* = 0.0001). This analgesic effect was durable, persisting through day 14 (*P* = 0.0085), day 21 (*P* < 0.0001), and day 28 (*P* = 0.0022). Consistent with the VFT results, Dex/Lid@PLX/HA showed greater analgesic efficacy than the other delivery formats by day 28, prolonging latency by 6.07 s compared to Dex/Lid@PBS (*P* = 0.0079) and by 4.52 s compared to Dex/Lid@PLX (*P* = 0.0091).

To assess the therapeutic efficacy of the hydrogel formulations against neuropathic mechanical hypersensitivity, the VFT was conducted over 28 d (Fig. 3C). As anticipated, CCI induction precipitated a significant reduction in PWTs by day 1 [Naive versus Injury: mean diff. = 33.42 g, 95% confidence interval (CI): 22.42 to 44.41, *P* = 0.0003], establishing a baseline of robust mechanical allodynia. Among the treatment groups, Dex/Lid@PLX/HA demonstrated the earliest and most sustained therapeutic benefit. Significant elevation in PWT was observed as early as day 3 (mean diff. = 7.16 g versus Injury, *P* = 0.0105) and maintained through day 28 (*P* = 0.0003). Notably, at the late-stage endpoint (day 28), Dex/Lid@PLX/HA significantly outperformed both the solution-based Dex/Lid@PBS (mean diff. = 6.42 g, *P* = 0.0029) and the standard gel Dex/Lid@PLX (mean diff. = 5.13 g, *P* = 0.0056). These data suggest that the HA-enhanced network not only prolongs drug bioavailability but effectively arrests the maintenance of mechanical allodynia.

Collectively, these behavioral results highlight the distinct advantage of the Dex/Lid@PLX/HA formulation. While Dex/Lid@PBS failed to provide significant analgesia and Dex/Lid@PLX offered only transient relief that diminished after the subacute phase, the HA-enhanced system provided sustained analgesic benefit against both mechanical and thermal sensitization. This sustained efficacy supports the hypothesis that HA incorporation optimizes local drug retention to effectively counter the chronification of neuropathic pain.

### Histological evidence of peripheral neuroprotection in sciatic nerve and DRG

To evaluate structural preservation, H&E staining was performed on sciatic nerve tissue (Fig. 3D). The Naive group displayed a coherent nerve architecture characterized by tightly packed, myelinated axons and minimal cellular infiltration. In contrast, the Injury group exhibited severe pathological changes consistent with Wallerian degeneration, including disordered axonal arrangement, extensive vacuolation, perineurial thickening, and marked infiltration of inflammatory cells. While Dex/Lid@PBS and Dex/Lid@PLX treatments conferred only marginal structural improvements with persistent inflammation, Dex/Lid@PLX/HA treatment showed better histological preservation than the PBS and PLX groups. This group maintained near-normal fiber organization with significantly reduced inflammatory infiltration, suggesting that the HA-enhanced hydrogel effectively stabilizes the nerve microenvironment to arrest degenerative cascades.

Parallel histopathological analysis of the DRG (Fig. 3E) revealed critical neuroprotective effects at the soma level. Naive DRGs featured healthy sensory neurons ensheathed by a thin, uniform layer of SGCs. Conversely, CCI injury precipitated neuronal stress, evidenced by neuronophagia, SGC hypertrophy, and the prominent formation of Nageotte nodules, residual glial clusters marking neuronal death. Treatment with Dex/Lid@PBS failed to arrest these degenerative changes. While Dex/Lid@PLX provided moderate preservation, Dex/Lid@PLX/HA showed the greatest degree of histological preservation among the tested groups, maintaining neuronal integrity and effectively suppressing the formation of Nageotte nodules and SGC vacuolization.

### Attenuation of macrophage infiltration and increase in CD163-positive reparative macrophage signals in the injured sciatic nerve

**Fig. 4.**
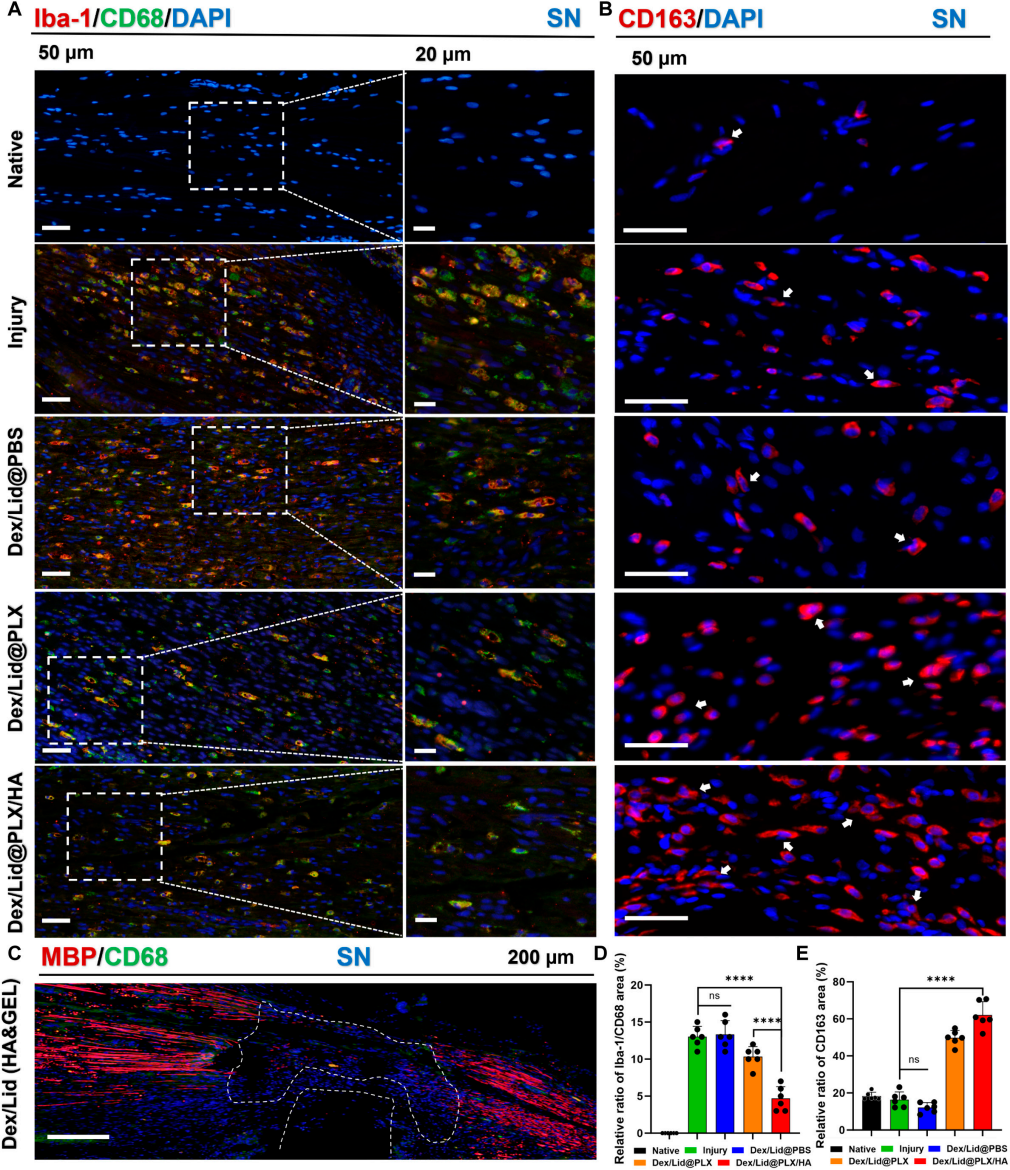
Dex/Lid@PLX/HA modulates macrophage-associated inflammatory markers and is associated with an increased CD163-positive macrophage signal in the injured sciatic nerve. (A) Representative immunofluorescence images of sciatic nerve sections stained for Iba-1 (red; pan-macrophage marker), CD68 (green; proinflammatory M1 marker), and DAPI (blue). Dex/Lid@PLX/HA treatment significantly suppresses the infiltration of double-positive (yellow) M1 macrophages. Scale bars, 50 μm (left) and 20 μm (right). (B) Immunofluorescence staining for CD163 (red; marker associated with reparative macrophages) and DAPI (blue). Dex/Lid@PLX/HA exhibits the highest density of CD163^+^ cells (white arrows), indicating increased CD163-positive reparative macrophage-associated signal. Scale bar, 50 μm. (C) Representative immunofluorescence image showing the preservation of myelin basic protein (MBP; red) and reduction of CD68^+^ macrophages (green) in the Dex/Lid@PLX/HA group. The dashed line outlines the nerve structure. Scale bar, 200 μm. (D) Quantitative analysis of the relative Iba-1^+^/CD68^+^ positive area (%) within the sciatic nerve region. (E) Quantitative analysis of the relative CD163^+^ positive area (%) within the sciatic nerve region. Data are presented as mean ± SD. ****P < 0.0001, ***P < 0.001, **P < 0.01, ns: not significant.

To elucidate the immunomodulatory mechanism of the hydrogel system, we analyzed macrophage phenotypic dynamics in the sciatic nerve at 28 d post-injury (Fig. 4). We quantified the expression of Iba-1^+^/CD68^+^ cells (proinflammatory M1 phenotype) and CD163^+^ cells (anti-inflammatory/reparative M2 phenotype).

As shown in Fig. 4A and D, CCI injury triggered a robust inflammatory response characterized by a significant infiltration of Iba-1^+^/CD68^+^ macrophages compared to the Naive group (mean diff. = −13.04, *P* < 0.0001). This M1-dominant profile remained largely unchanged in the Dex/Lid@PBS group, indicating that transient drug exposure failed to resolve chronic inflammation. While Dex/Lid@PLX treatment partially attenuated M1 activation (mean diff. = 2.71 versus Injury, *P* = 0.027), it significantly reduced M1 marker expression (mean diff. = 8.37 versus Injury, *P* < 0.0001), restoring levels closer to the Naive baseline (mean diff. = −4.67, *P* < 0.0001). Crucially, we observed a distinct shift in macrophage polarization toward the reparative phenotype (Fig. 4B and E). Dex/Lid@PLX/HA treatment increased the density of CD163^+^ M2 macrophages (mean diff. = −45.68 versus Injury, *P* < 0.0001), significantly outperforming both Dex/Lid@PLX (mean diff. = −12.33, *P* = 0.0005) and Dex/Lid@PBS (mean diff. = −49.99, *P* < 0.0001). This finding suggests that the HA-containing formulation may contribute to a macrophage profile consistent with reparative polarization in the injured sciatic nerve. However, the present study did not directly test whether this effect was mediated by CD44-dependent signaling.

To correlate this immunomodulation with structural neuroprotection, we performed double immunofluorescence staining for myelin basic protein (MBP) and CD68 (Fig. 4C). In the Dex/Lid@PLX/HA group, preserved myelin architecture was observed in parallel with minimal macrophage infiltration. This spatial association suggests that the reduced inflammatory burden and increased CD163-positive macrophage signal observed in the HA-enhanced hydrogel group were accompanied by better preservation of myelin integrity.

### Suppression of TRPV1-mediated sensitization in DRG and spinal dorsal horn

**Fig. 5.**
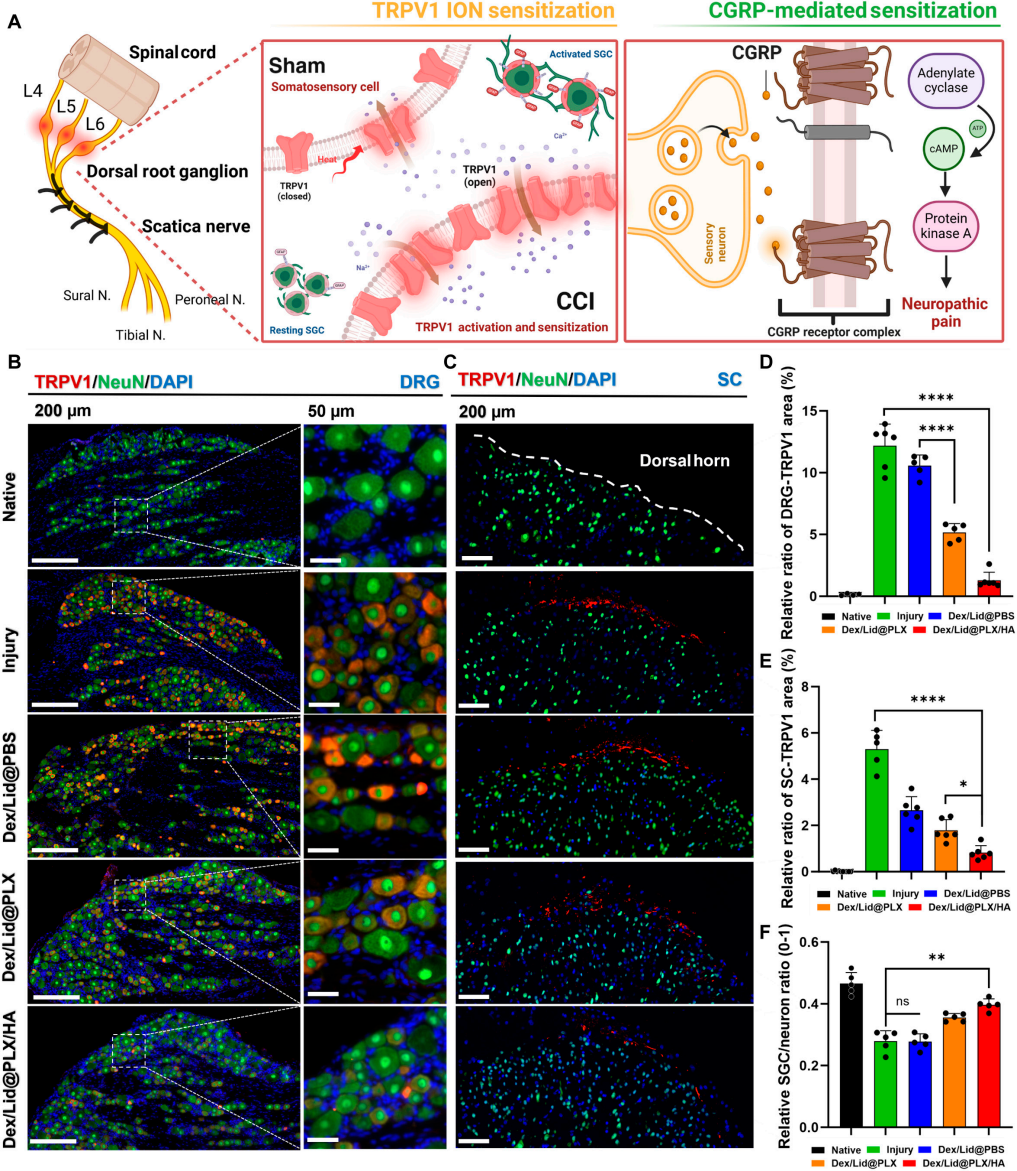
TRPV1-mediated nociceptive sensitization in the DRG and spinal cord dorsal horn (SC) following CCI and treatment. (A) Schematic illustration of the proposed mechanism: Injury-induced TRPV1 ion channel activation triggers calcium influx and downstream CGRP release, which activates adenylate cyclase/PKA signaling to amplify neuropathic pain sensitization. (B) Representative immunofluorescence images of the DRG stained for TRPV1 (red), NeuN (green), and DAPI (blue). The Injury group shows marked up-regulation of TRPV1 in sensory neurons. Dex/Lid@PLX/HA treatment substantially reduces TRPV1 expression, restoring it to near-Naive levels. Scale bars, 200 μm (overview) and 50 μm (inset). (C) Immunofluorescence staining for TRPV1 (red) and NeuN (green) in the spinal dorsal horn. The dashed line indicates the dorsal horn boundary. Dex/Lid@PLX/HA significantly suppresses injury-induced central TRPV1 up-regulation. Scale bar, 200 μm. (D to F) Quantitative analysis of the relative TRPV1^+^ area in the DRG (top) and spinal cord (middle), and the SGC/neuron ratio. Dex/Lid@PLX/HA shows marked suppression of TRPV1 overexpression relative to the injury group. Data are presented as mean ± SEM. ****P < 0.0001, **P < 0.01, *P < 0.05, ns: not significant.

To investigate the impact of our delivery strategy on nociceptive sensitization, we evaluated TRPV1 expression in the DRG and spinal dorsal horn, key sites orchestrating peripheral and central pain processing (Fig. 5A). In the Naive group, TRPV1 expression in NeuN^+^ sensory neurons was minimal, with surrounding SGCs displaying a quiescent morphology (Fig. 5B). However, CCI injury triggered a marked up-regulation of TRPV1^+^ neurons in both the DRG (mean diff. = −12.05 versus Naive, *P* < 0.0001) and the spinal dorsal horn (mean diff. = −5.29, *P* < 0.0001; Fig. 5C), consistent with hyperexcitable afferent signaling and central sensitization.

This pathological TRPV1 up-regulation persisted in the Dex/Lid@PBS group (DRG: *P* < 0.0001 versus Naive), suggesting that transient free drug diffusion is insufficient to reverse the sensitization cascade. Mechanistically, as illustrated in Fig. 5A, persistent TRPV1 activation likely promotes excessive Ca^2+^ influx and engages downstream CGRP-dependent signaling via the adenylate cyclase–PKA pathway, thereby reinforcing neuron–glia crosstalk and amplifying pain transmission.

In contrast, the hydrogel-based systems, particularly Dex/Lid@PLX/HA, produced a progressive reduction of TRPV1 expression. Quantitatively (Fig. 5D), Dex/Lid@PLX/HA restored TRPV1 levels to near-Naive baseline in the DRG (mean diff. = −1.15 versus Naive, ns) and significantly suppressed spinal TRPV1 expression (Fig. 5E; mean diff. = −0.81, *P* = 0.0208). Crucially, when compared directly with Dex/Lid@PLX, the HA-enhanced formulation provided significantly greater inhibition of TRPV1 overexpression [DRG: mean diff. = −3.88, *P* < 0.0001; spinal cord dorsal horn (SC): mean diff. = −0.97, *P* = 0.0106], aligning with its greater behavioral analgesic effect. The fact that TRPV1 down-regulation in the DRG paralleled that in the spinal dorsal horn is consistent with reduced central nociceptive signaling associated with effective peripheral desensitization.

Furthermore, analysis of the neuron–glia unit integrity revealed that while injury caused a significant drop in the SGC coverage of neurons, Dex/Lid@PLX/HA treatment reversed this alteration. To quantify this pathological glial reactivity, we calculated the SGC/neuron area ratio. As expected, the Injury group exhibited a sharp increase in this ratio compared to the Naive group (mean diff. = 0.1865, *P* < 0.0001), indicative of robust SGC activation. Dex/Lid@PBS treatment failed to significantly reduce this ratio (*P >* 0.05), indicating that transient drug exposure is insufficient to quell gliosis. In contrast, Dex/Lid@PLX/HA treatment significantly attenuated SGC activation (mean diff. = −0.1178 versus Injury, *P* < 0.0001), restoring the ratio to levels comparable to the Naive state (Fig. 5F). Pairwise comparisons further confirmed that Dex/Lid@PLX/HA was significantly more effective than both Dex/Lid@PBS (*P* < 0.0001) and Dex/Lid@PLX (mean diff. = −0.0414 versus PLX).

Collectively, these findings confirm that the sustained perineural co-delivery system not only preserves the homeostatic neuro-glial environment and dampens pathological neuron–glia coupling but also successfully blunts TRPV1-driven hyperexcitability and disrupts the CGRP-mediated positive feedback loop between peripheral and central nociceptive circuits.

### Inhibition of glial activation and CGRP-mediated neuron–glia crosstalk in the DRG

**Fig. 6.**
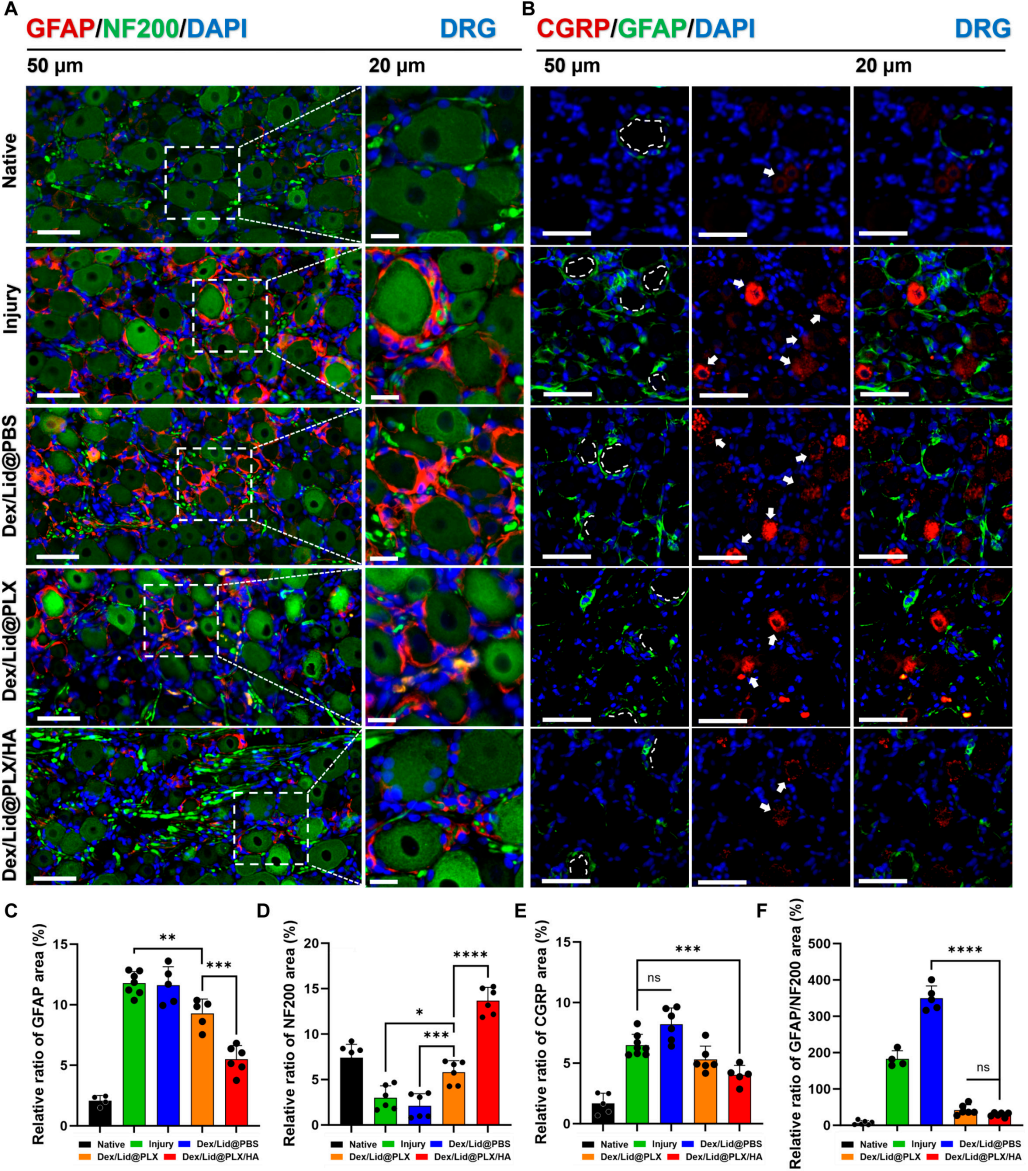
Dex/Lid@PLX/HA attenuates satellite glial activation and CGRP-mediated nociceptive signaling in the DRG. (A) Representative immunofluorescence images of DRG stained for GFAP (red; SGC marker), NF200 (green; myelinated neuron marker), and DAPI (blue). The Injury and PBS groups display marked SGC activation (gliosis) and neuronal atrophy. Dex/Lid@PLX/HA treatment suppresses GFAP expression and restores robust NF200 staining, indicating neuroprotection. Scale bars, 50 μm (left) and 20 μm (right). (B) Immunofluorescence staining for CGRP (red; nociceptive peptide), GFAP (green), and DAPI (blue). White arrows highlight clusters of reactive SGCs surrounding CGRP-high neurons. Dex/Lid@PLX/HA significantly reduces CGRP intensity and prevents SGC clustering. Scale bars, 50 μm. (C to F) Quantitative analysis of (C) GFAP-positive area, (D) NF200-positive area, (E) CGRP-positive area, and (F) the GFAP/NF200 ratio. Compared to the control groups, Dex/Lid@PLX/HA exhibited a significantly greater reduction in gliosis and neuropeptide release while simultaneously preserving neuronal structure. Data are presented as mean ± SD. ****P < 0.0001, ***P < 0.001, **P < 0.01, *P < 0.05, ns: not significant.

To evaluate the impact of treatment on the neuro-glial unit, we co-labeled DRG sections for the neuronal cytoskeletal marker NF200 and the SGC marker GFAP (Fig. 6A). In the Naive group, GFAP expression was minimal, restricted to a thin, quiescent sheath around NF200-rich neurons. CCI induction precipitated massive SGC activation (gliosis), indicated by a sharp rise in GFAP expression (mean diff. = −9.71 versus Naive, *P* < 0.0001) and a concurrent loss of NF200 signal (mean diff. = −4.22 versus Naive, *P* < 0.0001), reflecting severe neuronal stress and structural disruption.

While Dex/Lid@PBS treatment failed to arrest this pathology—resulting in persistent gliosis and axonal loss, Dex/Lid@PLX/HA treatment achieved marked restoration of the neuro-glial architecture. It significantly suppressed GFAP levels (−7.22 versus Naive, *P* < 0.0001) and restored NF200 expression to levels significantly higher than the injury group (+4.48, *P* < 0.0001). Interestingly, the NF200 signal intensity in the Dex/Lid@PLX/HA group slightly exceeded baseline levels (Fig. 6D). This likely reflects a robust regenerative response during the recovery phase, where surviving neurons undergo cytoskeletal reorganization and axonal re-expansion following the successful resolution of acute inflammation.

We further interrogated the neuropeptidergic signaling axis by quantifying CGRP expression relative to SGC activation (Fig. 6B). Under physiological conditions, CGRP expression is low. Following CCI, CGRP levels surged (mean diff. = −4.81 versus Naive, *P* < 0.0001), signifying heightened nociceptor excitability. This up-regulation was exacerbated in the Dex/Lid@PBS group, indicating that transient drug delivery fails to block neuropeptide release. In marked contrast, Dex/Lid@PLX/HA significantly attenuated CGRP overexpression (mean diff. = −2.47 versus Injury, *P* = 0.0028), outperforming the Dex/Lid@PLX group (ns versus Injury). Quantitative analysis (Fig. 6E and F) confirmed that the HA-enhanced formulation effectively decouples the pathological crosstalk between hyperexcitable neurons and reactive SGCs, thereby dampening the peripheral drive for neuropathic pain maintenance.

The sol-to-gel transition observed in the hydrogel groups is attributed to the thermosensitive nature of PLXs, enabling the formulations to be readily injected as a sol and subsequently form a solid-like depot at physiological temperatures. This in situ gelation capability is critical for prolonging the residence time of the formulation in vivo [32]. The structural analysis indicates that the highly porous network observed in Dex/Lid@PLX/HA results from the cross-linked structure of the HA gel, which generates additional voids and channels within the PLX matrix. This open microstructure may facilitate greater water penetration and diffusion pathways [33,34]. Importantly, rheological data confirm that HA blending modifies the internal architecture without compromising the gel-like viscoelastic nature of the system, as evidenced by the elastic-dominant behavior (*G*′ > *G*″) [35,36]. Furthermore, the low tan δ values, characteristic of stable hydrogels maintaining solid-like behavior under physiological conditions, suggest that HA blending modifies the internal architecture while retaining the system’s viscoelastic properties [37]. The drug release kinetics highlight the role of the hydrogel matrix as a diffusion barrier. The slower release observed in the HA-containing hydrogel is likely due to the concentration-dependent nature of PLXs and the altered network density upon HA incorporation, which effectively enhances the sustained-release characteristics of the system [33,38].

In terms of safety, the biocompatibility assessment suggests that the functional hydrogels are suitable as drug delivery systems. Although a slight reduction in cell viability was observed in drug-treated groups, likely due to the mild pharmacological activity of the encapsulated agents, the maintenance of viability above 80% and the unimpaired cell migration indicate acceptable biocompatibility without functional cytotoxicity. Furthermore, the formulations demonstrated significant cytoprotective effects against TNF-α-induced apoptosis. The successful reversal of proapoptotic and anti-apoptotic gene expression trends in all drug-treated groups confirms that the hydrogel matrix allows for the effective release of therapeutic agents without compromising their biological activity. It is noteworthy that no significant difference in therapeutic efficacy was observed between the sustained-release hydrogel groups and the bolus PBS group within the short 24-h timeframe of the cell-based assay. This suggests that the 24-h cell-based assay was sufficient to confirm preservation of immediate drug bioactivity, but was not adequate to capture formulation-dependent advantages related to local retention and sustained release that became more evident in vivo.

An explanation for the more limited separation among formulations in vitro than in vivo is that the cell-based assays were designed primarily to confirm short-term drug bioactivity under simplified conditions, rather than to reproduce the pharmacokinetic and multicellular complexity of the injured nerve microenvironment. In the 24-h SH-SY5Y model, all formulations were able to expose cells to biologically active Dex and Lid over a short diffusion distance, which reduced formulation-dependent differences. In contrast, the in vivo CCI model incorporates additional determinants of efficacy, including local perineural retention, rapid clearance of the solution formulation, sustained drug release from the gel depot, repeated inflammatory stimulation, and dynamic interactions among neurons, satellite glial cells, and infiltrating immune cells over days to weeks. Therefore, the in vitro results mainly demonstrate preservation of immediate drug activity, whereas the in vivo results more effectively reveal the therapeutic advantage of prolonged local delivery by the PLX/HA system.

Next, the more pronounced anti-inflammatory performance of the Dex/Lid@PLX/HA formulation, particularly in suppressing COX-2 and IL-6, underscores the importance of the delivery vehicle’s structural properties. The failure of the PBS and simple Dex/Lid@PLX groups to significantly reduce COX-2 levels suggests that transient drug exposure may be insufficient to quell persistent inflammatory signaling. In contrast, the significant down-regulation achieved by the Dex/Lid@PLX/HA group indicates that the addition of HA contributes to improved local retention and controlled drug availability. This is likely achieved by HA enhancing the localized retention and controlled availability of the drugs against the inflammatory cascade, aligning with recent strategies in spatiotemporal delivery control [39]. In the SH-SY5Y inflammatory stress model, the Dex/Lid@PLX/HA group showed a more moderate IL-4 transcriptional response than the PBS and PLX groups. However, because IL-4 was measured in a neuronal cell context rather than in immune cells, this observation should be interpreted cautiously and not taken as direct evidence of macrophage polarization. These findings highlight the potential of the Dex/Lid@PLX/HA system as a clinically relevant platform for treating chronic inflammatory neuropathies.

Crucially, the translation of these material advantages into in vivo efficacy is evidenced by the sustained analgesic effects over the 28-d observation period. Although the present study did not include Dex- or Lid-only treatment arms, the 2 agents were selected to address complementary components of neuropathic pain pathology. Dex likely contributed primarily to suppression of the local inflammatory cascade, consistent with the reductions in NF-κB/COX-2/IL-6-associated signaling and inflammatory cell infiltration. In contrast, Lid likely contributed mainly to the attenuation of ectopic nociceptive activity and peripheral sensitization through sodium channel blockade, which is consistent with the improvement in behavioral hypersensitivity and the reduction of TRPV1/CGRP-associated nociceptive signaling. Accordingly, the current data are best interpreted as supporting a complementary anti-inflammatory and analgesic co-delivery strategy rather than assigning exclusive effects to either agent.

Unlike free drug administration, which suffers from rapid clearance, the Dex/Lid@PLX/HA system provided sustained relief from mechanical allodynia and thermal hyperalgesia for up to 28 d. This performance is linked to the suppression of the TRPV1–CGRP–SGC axis. Our data show that sustained local delivery effectively down-regulates TRPV1 expression in both the DRG and spinal dorsal horn. Since TRPV1 activation drives calcium influx and the release of CGRP, its suppression disrupts the positive feedback loop of neurogenic inflammation. Consequently, we observed a marked reduction in CGRP levels and the attenuation of SGC activation in the DRG. By preventing the formation of Nageotte nodules and interrupting pathological neuron–glia crosstalk, the hydrogel addresses the root causes of central sensitization rather than merely masking symptoms.

Furthermore, the immunomodulatory capacity of the system extends beyond simple anti-inflammation to active immune reprogramming. HA has been reported to interact with receptors such as CD44 on immune cells, and certain HA-based biomaterials have been associated with reduced inflammatory signaling and a shift toward more pro-regenerative macrophage phenotypes in other disease or tissue-repair settings. In the present study, the increased CD163-positive macrophage signal observed in the Dex/Lid@PLX/HA group is therefore consistent with, but does not by itself prove, a contribution of HA-related immunomodulation. Direct verification of CD44 involvement will require dedicated experiments, such as receptor-blocking studies or macrophage-specific in vitro assays [40]. M2 macrophages are essential for debris clearance and the secretion of neurotrophic factors, thereby creating a permissive environment for nerve regeneration [41]. This immune-instructive property, coupled with the neuroprotective preservation of myelin architecture, underscores the Dex/Lid@PLX/HA platform as a therapeutic strategy that integrates sustained pharmacology with bioactive material design.

While this study establishes the efficacy of the PLX@HA platform for localized co-delivery, several limitations warrant consideration. Primarily, because monotherapy arms were not included, the individual contributions of Dex and Lid could not be strictly distinguished in the present study. Their respective roles are therefore interpreted on the basis of known pharmacology together with the observed inflammatory and nociceptive readouts. Furthermore, head-to-head comparisons with systemic standard-of-care regimens were not performed in this proof-of-concept evaluation. Future investigations will therefore employ noninferiority designs to benchmark the PLX@HA platform against clinical standards and quantify pharmacological synergy. Additionally, the 28-d observation period effectively captured the subacute-to-chronic transition but precluded the assessment of long-term durability and safety. Given the potential for steroid-induced local tissue atrophy or fibrosis upon prolonged exposure, future follow-up should be extended to 8 to 12 weeks. Subsequent studies will incorporate systemic toxicity panels and broader functional endpoints to definitively rule out late-onset adverse effects while confirming the persistence of analgesia and structural regeneration.

## Conclusion

This study shows that localized co-delivery of Dex and Lid using an injectable PLX/HA hydrogel provides sustained analgesic benefit in a rat CCI model. The treatment was associated with improved behavioral outcomes, reduced inflammatory and nociceptive marker expression, and preservation of peripheral nerve and DRG histological features. These findings support the potential of the PLX/HA platform as a promising local delivery strategy for neuropathic pain, while further mechanistic, pharmacological, and long-term safety studies are still required.

## Data Availability

All data needed to evaluate the conclusions in the paper are present in the paper.
